# Inflammatory-myofibroblastic tumour with acute calculous cholecystitis in a sessile gall bladder in a young woman

**DOI:** 10.1093/jscr/rjag482

**Published:** 2026-07-11

**Authors:** Venkiteswaran Muralidhar, Ram Prasath, Govindasamy Prabhu, Jyothi Ravuri

**Affiliations:** Bioinformatics, University of Birmingham, Birmingham, United Kingdom; Department of Surgery, Chettinad Academy of Research and Education, Kelambakkam, Chennai 600103, India; Department of Surgery, Chettinad Academy of Research and Education, Kelambakkam, Chennai 600103, India; Department of Surgery, Chettinad Academy of Research and Education, Kelambakkam, Chennai 600103, India

**Keywords:** gallbladder tumour, inflammatory, myofibroblastic

## Abstract

A woman in her 30s presented with acute cholecystitis. Blood and radiological investigations supported the clinical diagnosis. Diagnostic laparoscopy showed a thickened inflamed gall bladder (GB). Laparotomy was performed that showed a thickened sessile inflamed GB. There was no involvement of adjacent organs, ascites or metastasis. Histopathology showed an inflammatory myofibroblastic tumour (IMT) in a sessile GB. The tumour is classified as intermediate grade of malignancy by the World Health Organization. There are eight reported cases of IMT, all of which were successfully treated with resectional surgery with only one recurrence at 13 months. One year later, our case had no evidence of recurrence. To conclude IMT could be successfully treated by open surgical resection. To the best of our knowledge, this is the first report of an IMT of the GB presenting with acute calculous cholecystitis; in addition, the cystic duct was absent.

## Introduction

Inflammatory myofibroblastic tumour (IMT) of the gallbladder (GB) is a rare tumour, with only eight cases published in the literature [1, 2]. The IMT of the GB is classified as an intermediate-grade malignancy [3], can be locally aggressive, and metastasis has not been reported. The aetiology and treatment of IMT of the GB require clarification. The diagnosis of IMT of the GB is made by histopathology and could harbour genomic variations [4]. The sessile GB is the rarest of the anatomical variations in the insertion of the cystic duct onto the common bile duct (CBD) [5, 6].

## Case presentation

A woman in his 30s, with no prior morbidities, presented with acute right hypochondrial pain with vomiting over 2 days. She had no relevant medical comorbidities in the past. She had a pulse of 120 beats per minute, blood pressure of 140/80 mm of mercury, respiratory rate of 28 per minute and a Glasgow Coma Scale of 15. There was fullness in the right hypochondrium with a positive Murphy’s sign. There was no other abdominal tenderness, and the hernial orifices were normal. Chest examination was normal. There was no lymphadenopathy. The skull and spine were normal. There was nothing significant on a rectal examination.

An X-ray of the chest showed normal findings. Blood investigations showed haemoglobin 85 g/L, total leucocyte 7400/μl, serum creatinine 0.82 mg/dl, random blood sugar 106 g/dl, alanine transaminase 44 U/L, aspartate transaminase 56 U/L, alkaline phosphatase 55 U/L, and total bilirubin 0.8 mg/dl. Ultrasonography showed calculous cholecystitis; the CBD was not identified. Oesophagogastroduodenoscopy was normal. A magnetic resonance cholangiopancreatogram (MRCP) showed a thickened GB with a lateral indentation of the CBD (Fig. 1).

**Figure 1:**
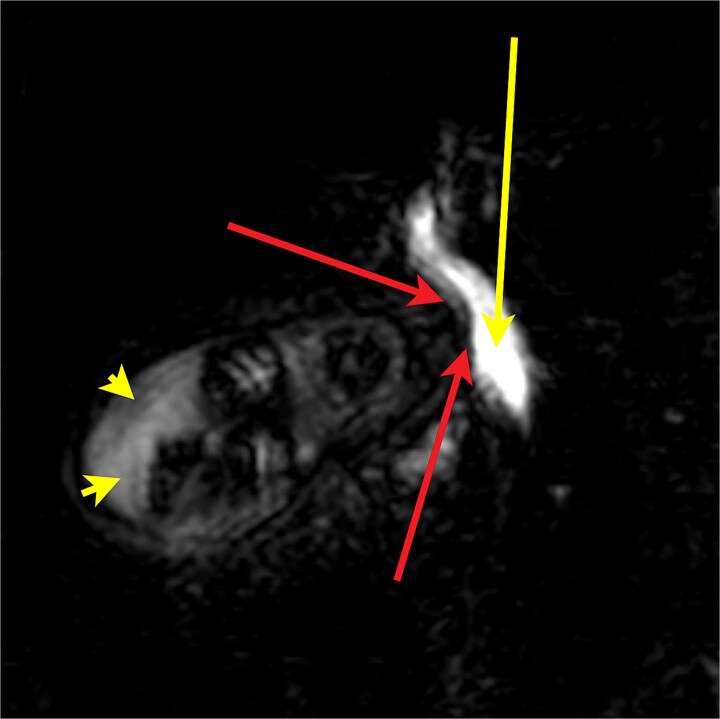
A MRCP image showing the indentation (red arrows) of the common bile duct (yellow arrow) from a thickened sessile gall bladder, thickened fundus of the gall bladder (yellow arrow heads).

### Differential diagnosis

1. Chronic cholecystitis
2. Cholesterolosis
3. Adenomyosis
4. Lymphoma

## Discussion

The diagnosis was acute cholecystitis and hence she underwent a diagnostic laparoscopy followed by open cholecystectomy on the second day after admission [6]. The GB wall was thickened throughout, measured two centimeters at the distal end and opened directly into the CBD (Figs 2 and 3). The opening on the lateral wall of the CBD was sutured with interrupted absorbable sutures. The patient developed a bile leak of > 150 ml per day; hence, her bile duct was stented. A repeat MRCP showed normal CBD. The patient was asymptomatic with absent bile leak on the seventh postoperative day and was discharged. She was asked to follow up for removal of the stent after six weeks. The histopathology of the specimen showed an.

**Figure 2:**
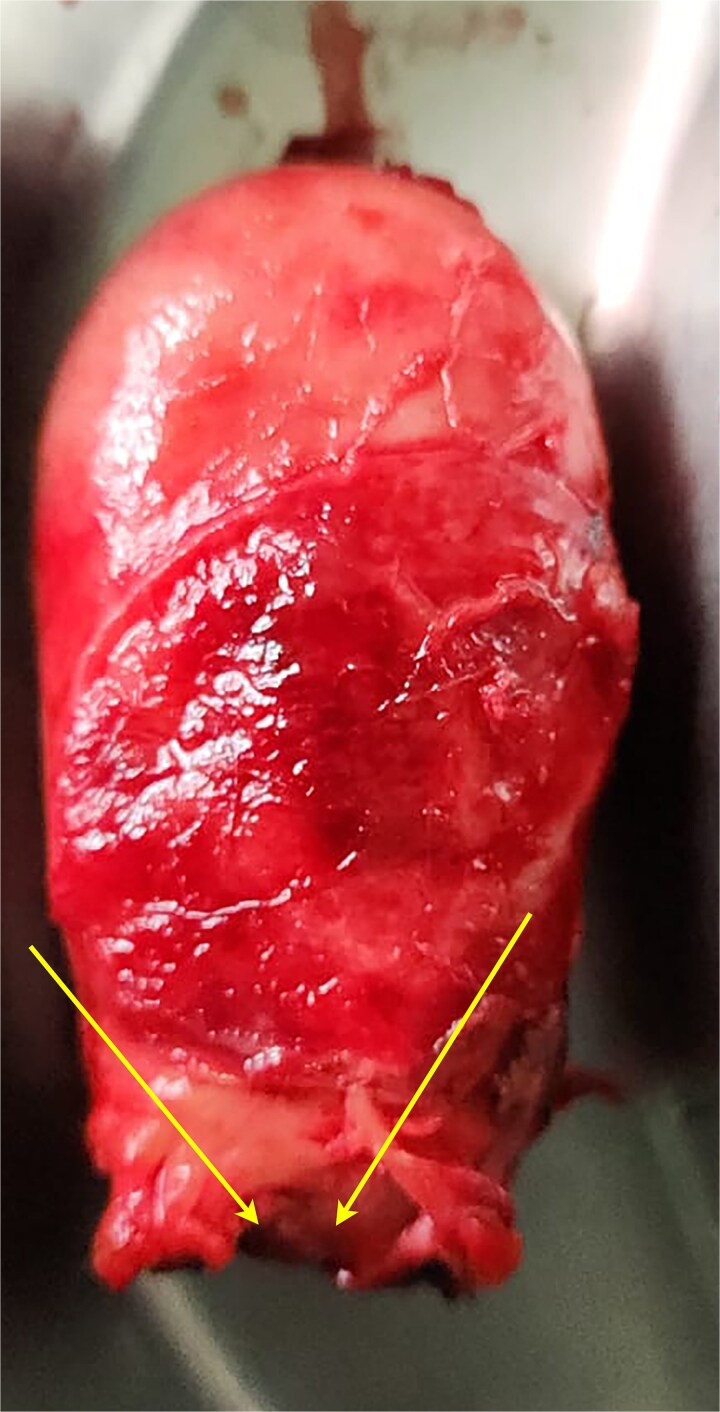
Specimen of the sessile gall bladder (yellow arrows show the distal end).

**Figure 3:**
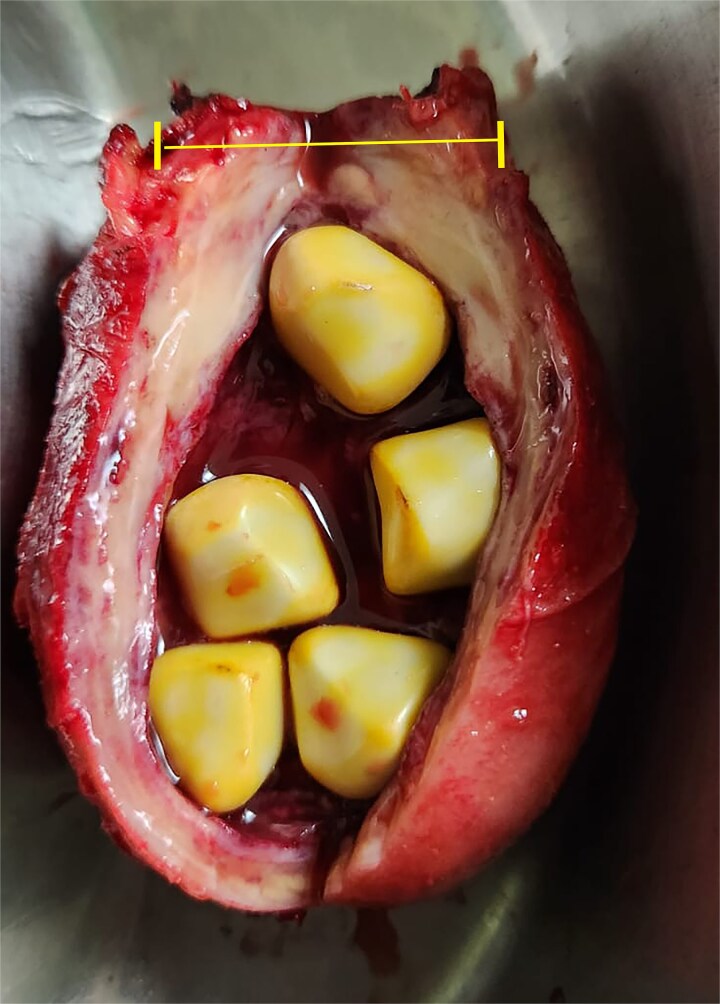
Specimen of the gall bladder with stones showing thickened wall, the distance at the distal end is 2 centimeters (yellow bracket).

The biliary stent was removed after 6 weeks. The patient was asymptomatic at one year follow up, ultrasonography and computed tomography scan showed no recurrence IMT of the GB with clear margins (Figs 4--7).

**Figure 4:**
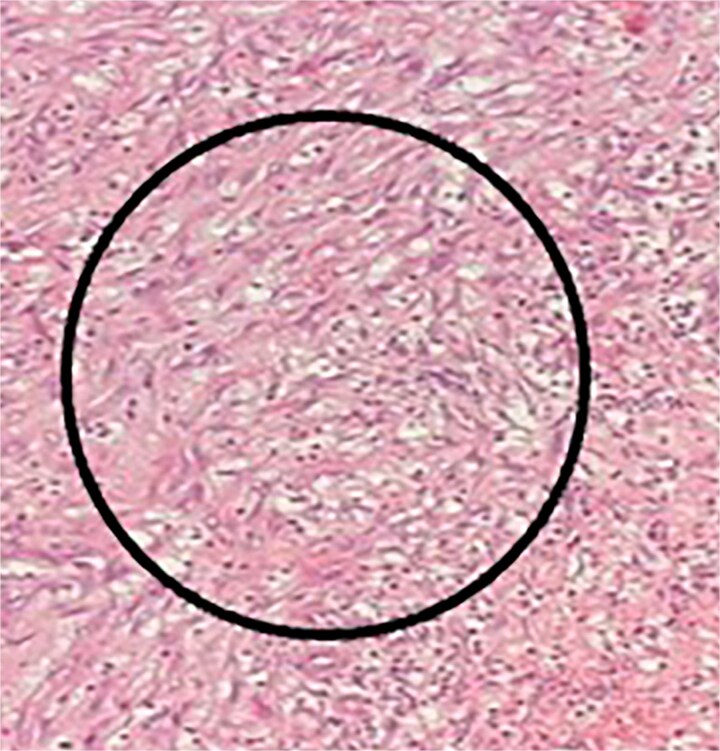
Histopathology 40× haemoxylin eosin stain showing myofibroblasts within the black circle.

**Figure 5:**
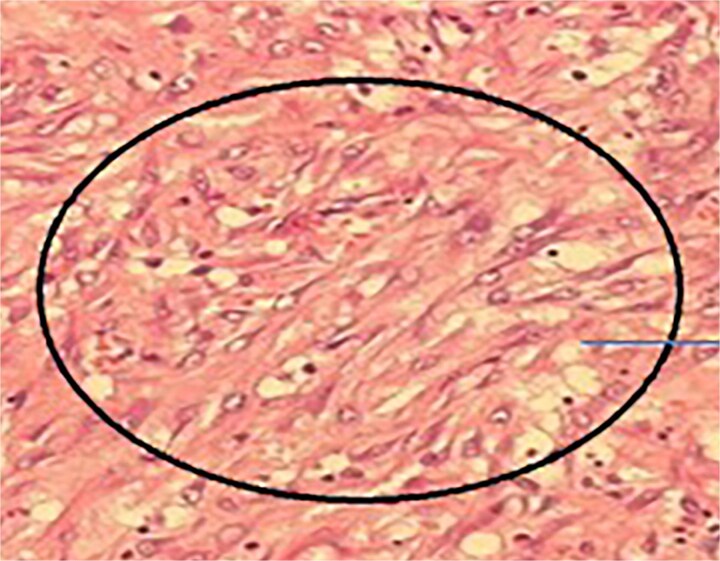
Histopathology 40× haemoxylin eosin stain showing storiform fibrosis with plump oval cells having prominent nucleoli and inflammatory cells (labelled within the image).

**Figure 6:**
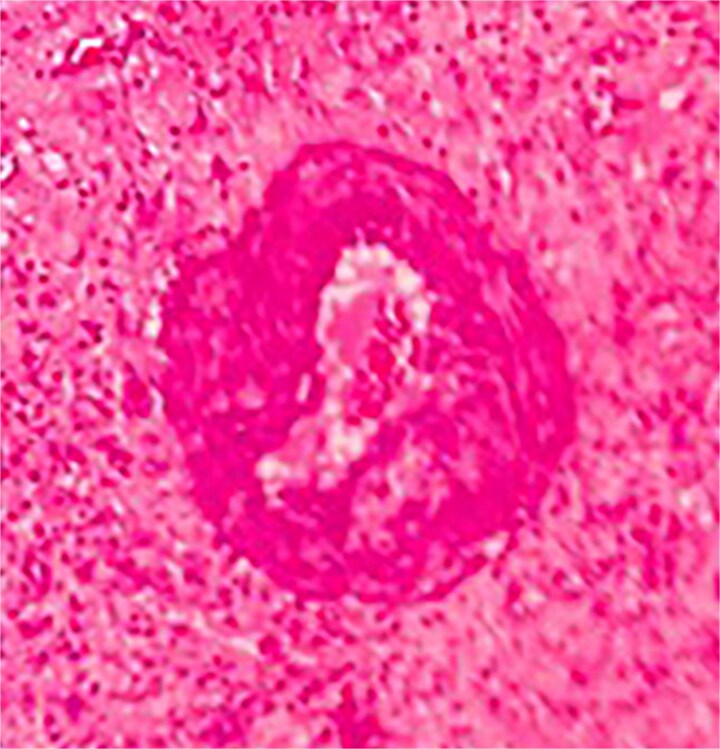
Histopathology 40× haemoxylin eosin stain showing thick hyalinized capillary wall.

**Figure 7:**
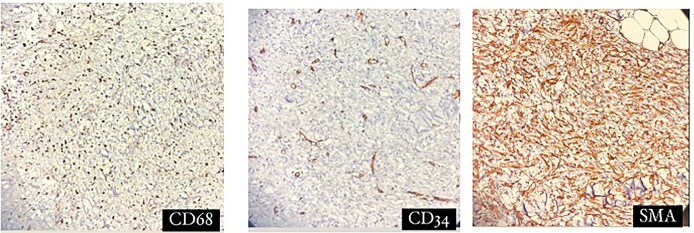
Immunohistochemistry for CD 68, CD34 and SMA as labelled in the respective images.

We report a rare case of acute calculous cholecystitis with a benign tumour in a sessile GB. IMT is a rare tumour (< 10 reported cases) of unknown aetiology, occurring in the age group of 30–35 [1]. The diagnosis is made based on classical histopathological features [4]. There have been eight case reports published of IMT of the GB [1]. These eight cases are tabulated (Table 1).

**Table 1:** Published cases of IMT of the gallbladder (modified from [1]).

Reference	Sex	Age	Preoperative diagnosis / Radiology findings	Surgery	Organ involved	Size (cm)	Outcome
Behranwala *et al.* [7]	F	51	Acute cholecystitis	Cholecystectomy, transverse colectomy	Gallbladder, transverse colon	12	Disease-free for 6 months
Muduly *et al.* [2]	F	35	Gallbladder cancer	Radical cholecystectomy	Gallbladder	NA	Disease-free for 2 years
Özsan *et al.* [8]	M	66	Gallbladder cancer	Radical cholecystectomy	Gallbladder	NA	NA
Badea *et al.* [9]	F	65	Gallbladder cancer	Radical cholecystectomy	Gallbladder, liver	6.5 × 5.2 × 4	Disease-free for 3 months
Sinha *et al.* [10]	F	36	NA	Radical cholecystectomy, resection of 1st part of the duodenum and pylorus.	Gallbladder, duodenum (1st part), pylorus	NA	NA
Maruyama *et al.* [11]	F	63	Gallbladder tumor	Cholecystectomy	Gallbladder	NA	Recurrence at 13 months
Yamada *et al.* [12]	M	50	gallbladder cancer	Cholecystectomy, liver resection	Gallbladder	2	Disease-free for 6 years
Losuwarat *et al.* [1]	M	63	Mass replacing gallbladder	Cholecystectomy, R hepatectomy, resection of the bile duct and the 2nd part of duodenum.	Gallbladder, liver, common bile duct, duodenum (2nd part).	9 × 7	Disease-free 2 yrs
Current study	F		Acute cholecystitis	Cholecystectomy	Gallbladder	12 × 5	Disease free 1 year

NA: Data not available.

Table 1 summarizes the nine published cases of GB IMT, including the present case, and highlights several consistent clinicopathological features. The condition predominantly affects middle-aged adults (35–66 years) with a female preponderance (7/9 cases). All reported patients were symptomatic, most commonly presenting with abdominal pain. In every instance, the preoperative diagnosis was GB carcinoma or cholecystitis, underscoring the strong clinical and radiological mimicry of malignancy. All patients underwent cholecystectomy, frequently with en bloc resection of adjacent organs, reflecting the locally infiltrative appearance of the tumour.

In most cases, disease involvement was confined to the GB with extension to a single neighbouring organ; only one report described multi-organ involvement, including the liver, bile duct, colon, and duodenum. Tumour size, when documented, ranged from 2 to 12 cm, with the lesion in the current case measuring 9 × 7 cm. Overall outcomes were favourable, with no metastatic disease reported and most patients remaining disease-free over follow-up periods ranging from 3 months to 6 years. A single case described non-resectable pancreatic recurrence at 13 months, which was refractory to steroids and non-steroidal anti-inflammatory drugs but showed regression with vinorelbine and methotrexate, suggesting a potential role for chemotherapy in selected recurrent cases. Collectively, these observations indicate that GB IMT is a rare entity that closely simulates malignancy, is definitively diagnosed only on postoperative histopathology, and is best managed with surgical resection, which is associated with excellent outcomes. Furthermore, given the limited number of reported cases, the prognostic significance of preoperative radiological findings remains uncertain.

IMT is regarded as being in the ‘grey zone’ between benign and malignant tumours due to its intermediate biological potential [13]. While it has a low risk of recurrence and metastatic potential, it is still classified as a neoplastic disease. This classification stems from its variable behaviour, which can range from indolent to aggressive, depending on factors such as genetic alterations (e.g. ALK rearrangements) and histological features [13]. It was only in 2020 that the WHO classified this tumour as a neoplasia with an intermediate grade [3]. There are no TNM staging criteria for this tumour, as IMT is a rare subset of soft-tissue sarcomas.

Furthermore, IMT of the GB is even rarer, with only eight cases reported (Table 1). Radical resection is not recommended for IMT of the GB. Authors of a case report of radical cholecystectomy performed for a suspected carcinoma of the GB suggested that if IMT could be diagnosed pre-operatively, then radical surgery might have been avoided [8]. Currently, the diagnosis of IMT of the GB is made after surgical treatment for a GB disease presenting as acute abdominal pain or as a suspected carcinoma (Table 1).

There are many variations in the insertion of the cystic duct into the CBD, the most common being an opening at an acute angle, and an absence of the cystic duct being the rarest (Fig. 8) [5].

**Figure 8:**
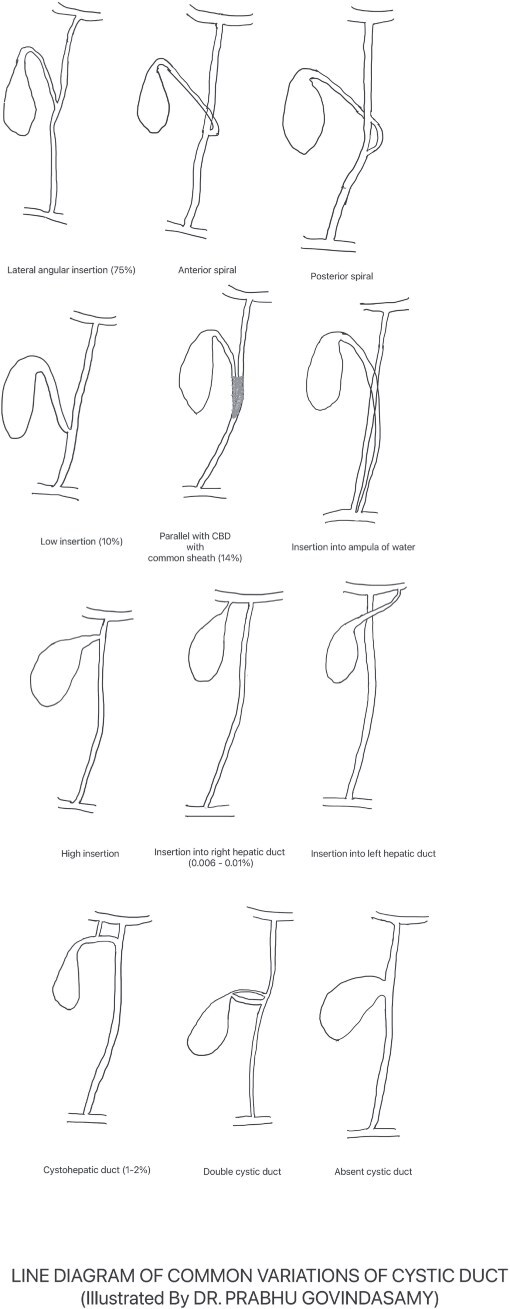
Line diagram showing the different insertions of the cystic duct (adapted from reference [1]).

## Learning points

- Myofibroblastic tumour of the GB can present acutely with calculous cholecystitis.
- The diagnosis of IMT of the GB can only be made post resection of the specimen.
- There is low recurrence after resection of IMT of the GB after surgical resection.

## References

[1] Losuwarat K, Luvira V, Thanasukarn V et al. Inflammatory myo-fibroblastic tumor of the gallbladder with multivisceral involvement: successful treatment with radical surgery. Case Rep Hepatol 2023;2023:1909570. 10.1155/2023/1909570 PMC10307192 37389088

[2] Muduly D, Deo SVS, Shukla NK et al. Inflammatory myofibroblastic tumor of gall bladder. Trop Gastroenterol 2012;33:297–9. 10.7869/tg.2012.79 23923363

[3] Sbaraglia M, Bellan E, Dei Tos AP. The 2020 WHO classification of soft tissue tumours: news and perspectives. Pathologica 2021;113:70–84. 10.32074/1591-951X-213 33179614 PMC8167394

[4] Gleason BC, Hornick JL. Inflammatory myofibroblastic tumours: where are we now? J Clin Pathol 2008;61:428–37. 10.1136/jcp.2007.049387 17938159

[5] Boushehry R, Husain F, Saleem A et al. Congenital absence of the cystic duct: case report of a rare anomaly and review of the literature. Int J Surg Case Rep 2022;96:107353. 10.1016/j.ijscr.2022.107353 35780648 PMC9284068

[6] Williams NS, Bullstrode CJK, O'Connell PR. Bailey & Love's Short Practice of Surgery. 25th ed: Annals of The Royal College of Surgeons of England 2010;92:178. 10.1308/rcsann.2010.92.2.178a

[7] Behranwala KA, Straker P, Wan A et al. Inflammatory myofibroblastic tumour of the gallbladder. World Journal of Surgical Oncology 2005;3:24. 15862123 10.1186/1477-7819-3-24 PMC1097763

[8] Özsan I, Özsoy M, Sahin E et al. Inflammatory myofibroblastic tumor of the gallbladder. Balkan Med J 2013;30:323–6. 10.5152/balkanmedj.2013.8263 25207130 PMC4115911

[9] Badea R, Veres AA, Andreica V et al. Inflammatory myofibroblastic tumor of the gallbladder: imaging aspects. J Med Ultrason 2015;42:89–95. 10.1007/s10396-014-0566-4 26578495

[10] Sinha L, Hasan A, Singh AK et al. Inflammatory myofibroblastic tumor involving liver, gallbladder, pylorus & duodenum: a rare case presentation. Int J Surg Case Rep 2017;31:27–9. 10.1016/j.ijscr.2016.12.017 PMC5238359 28092784

[11] Maruyama Y, Fukushima T, Gomi D et al. Relapsed and unresectable inflammatory myofibroblastic tumor responded to chemotherapy: a case report and review of the literature. Mol Clin Oncol 2017;7:521–4. 10.3892/mco.2017.1383 PMC5639331 29046787

[12] Yamada S. Atypical gallbladder cancer versus non‐neoplastic polypoid mass: inflammatory myofibroblastic tumor of the gallbladder – report. Clin J Gastroenterol 2018;11:437–42.

[13] Gros L, Dei Tos AP, Jones RL et al. Inflammatory myofibroblastic tumour: state of the art. Cancers (Basel) 2022;14. 10.3390/cancers14153662 PMC9367282 35954326

